# The Flavor of Dairy Products from Grass-Fed Cows

**DOI:** 10.3390/foods9091188

**Published:** 2020-08-27

**Authors:** Michele Faccia

**Affiliations:** Department of Soil, Plant and Food Sciences, University of Bari, Via Amendola 165/A, 70126 Bari, Italy; michele.faccia@uniba.it

**Keywords:** grass-based milk, dairy products, milk flavor, aroma-active compounds

## Abstract

The milks used for manufacturing bovine dairy products are not all equal. The feeding regimen of lactating cows can widely vary, giving rise to remarkable compositional differences. Recently, grass-fed/based milk and transformed products are being taken into great consideration due to their more favorable nutritional characteristics and better sustainability over those from intensive systems. Besides these well-established aspects, the existence of differences in flavor is highly debated. The “cheese story tellers” consider it as a proven fact and tend to directly link the aroma of grass-based dairy products to the plants the animals ate. Unfortunately, this claim is not yet supported by scientific data. Actually, there is sufficient evidence of the presence of a distinctive aroma in milk from grass-fed cows, but the connection with specific aroma-active compounds is still in progress. In addition to this, the role of some compounds deriving from cow’s metabolism seems to be much more important than that of other compounds that directly derive from feed. The situation in transformed products, in particular cheese, is even more complicated due to the overlapping of flavor compounds originating from technological operations, microbial metabolism and enzyme activities during storage or ripening. Further work is still needed to answer the question, but the increasing application of a flavoromics approach to the studies should rapidly bring about a decisive contribution to the knowledge.

Dairy products manufactured from milk of grass-fed cows are increasingly gaining the consumer’s approval for several reasons, such as healthier composition, better environmental sustainability and higher ethicality than its counterparts from intensive farming systems. In addition to this, many researchers and experts in food communication attribute them higher sensory quality, in particular a more pleasant flavor, considering it as a scientifically proven fact. Unfortunately, while the more favorable compositional, environmental and ethical aspects have been widely ascertained, those related to taste and aroma are still unclear. It depends on the fact that flavor formation in dairy products is the result of a series of factors and events that succeed and overlap each other with time: cattle feeding, management of milk at the farm, milk standardization and heat treatments at the dairy, microbial activities, technological treatments, proteolysis/lipolysis during refrigerated storage or during ripening of the products, etc. In particular, the flavor compounds arising from technological operations, microbial metabolism and enzyme activities tend to quickly overwhelm the weak flavor of milk, and any original sensory peculiarity tends to fade. Being not easy to isolate the effects of the single variables, any investigation aiming to assess the impact of the feeding regimen should be primarily conducted on fresh and simply processed products rather than on ripened or highly processed ones. Unfortunately, most of the data available in the literature are from studies on medium or long-ripened cheeses, whose results are often difficult to interpret [1,2,3,4]. To complicate things, a quantitative approach has been rarely applied in these studies. Such an approach is essential since the real impact of the potentially flavor-active compounds (both taste- and aroma-active) is closely related to the concentrations at which they are present in the product. Quantitative studies are difficult to perform for a series of reasons, the most important of which are mentioned below: (i) the most used analytical techniques (such as Solid Phase Micro-Extraction Gas Chromatography and some types of Liquid Chromatography, both coupled to Mass Spectrometry) mostly supply qualitative results, and shifting them into a “quantitative mode” is very laborious and time-consuming; (ii) complex experimental designs are required, involving flavoromics/sensomics approach with the support of olfactometry; (iii) dedicated model matrices must be developed, to be used as a basis for flavor compound recombination, addition of exclusion; (iv) the availability of a well-trained panel of experts for a long time is difficult and very expensive to obtain.

From the foregoing, it clearly appears that there is a long way still to go for fully understanding and defining in detail the impact of the feeding regimen on milk and cheese flavors. In this editorial article, we will mainly refer to aroma, which is one of the components of flavor, but is the most complex and most studied. In the case of fresh milk, aroma is the sensory characteristic that can better discriminate different types; the involved volatile compounds form the so-called “primary aroma”.

An excellent review on the flavor of grass-based milk and cheeses has been recently published by Kilcawley et al. [5]. Grass feeding can be conducted by keeping cattle on natural pastures or meadows for most of the day, as is the case in extensive farming systems, or by feeding them with fresh mowed grass. In both cases, the rumen metabolism and the connected mechanisms of milk synthesis are deeply affected, with important changes in the milk chemical composition. The most affected component is fat, whose level of saturation decreases, but other minor effects are also present, such as those on minerals and vitamins [6,7]. Besides the metabolic-mediated changes, grass feeding can be also responsible for direct transfer of some molecules from plants to milk, such as carotenoids, terpenes, aldehydes and ketones, alcohols and, possibly, phenols [5,8]. Under the sensory point of view, the yellow color and the smoother texture of derived butter and cheese is the most evident effect of grass feeding: these features are connected to the higher concentrations of β-carotene and unsaturated fatty acids with respect to non-grass-based products. However, what about aroma? For a long time, the researchers have almost exclusively paid attention to the role of the compounds directly derived from feed, in particular terpenes and their oxygenated derivatives, terpenoids. These isoprene-based molecules are contained in a number of spontaneous and cultivated plants, especially dicotyledonous species, and are transferred within a few hours into milk, where they tend to concentrate into the fat fraction due to their physico-chemical properties. However, as rapidly as they are transferred, rapidly do they disappear when the plant source is excluded from the cattle diet. Consequently, terpenes have been proposed as possible molecular markers of the type of feeding regimen and, to a certain extent, of the geographical origin (i.e., mountain versus valley farming) [9,10]; sometimes, they have been also indicated as being able to influence the flavor [11,12]. However, many researchers have questioned all these features and the view is going to change. In fact, it has been highlighted that the most terpenes cannot be aroma-active because of the high odor threshold with respect to the concentration in milk and cheese; furthermore, the effectiveness of their transfer to milk can be impaired by some molecular modifications that can be made by the rumen microflora [13,14,15]. The current consensus view is that an aromatic role can be played only if they are present at very high concentrations, as can happen in “strongly extensive” farming systems that leave cattle feeding (or, better to say, living) on natural pastures rich in spontaneous dicotyledonous plants. In recent years, the attention has been shifting to other substances, such as phenolic, hydrocarbon and heterocyclic compounds. It is known that phenols are powerful flavor-active molecules in a number of vegetables and vegetable-derived foods (i.e., wine and olive oil), in which they can be present at a level of several hundred milligrams kg^−1^. The presence of phenols in milk has long been neglected—but being widely ingested by grass-fed cattle, questioning about their presence and possible sensory role is fair. The evidence of their presence in milk, even though at low concentrations, is increasing, but the source and the mechanism by which they originate, and their impact on flavor, are still debating [16]. The main problem is the lack of reliable analytical techniques to extract and quantify them: effective isolation and chromatographic quantification is hindered by the complexity of the matrix and by the strong interactions with proteins; on the other hand, generic quantitative methods such as Folin–Ciocalteau assay and anti-oxidant activity tests can only be useful for comparative studies. Even though the transfer of some simple phenols from feed to milk has been sometimes reported, the most reliable results regard the presence of a phenol compound of metabolic origin, namely *p*-cresol, responsible for a barnyard-like and cowy odor. It derives from ruminal degradation of β-carotene and could be a key-aroma molecule of milk from grass-fed cows, in which the mother molecule is more abundant than in other feeding systems. Hydrocarbon and heterocyclic compounds are other molecules that could contribute to the peculiar taste of grass-based milk. Among them is toluene, another metabolite deriving from β-carotene degradation, but that can also derive from ruminal metabolism of the aromatic amino acids, tryptophan and tyrosine, when the ratio between crude protein and readily digestible carbohydrates is high [17]. Toluene odor has been described as almond, caramel, mothball, plastic. Other potentially flavor-active compounds in milk from grass-based farming systems are indole and skatole (presenting flowery of fecal odor, depending on the concentration): they also can originate from protein degradation in the rumen, and then are transferred to milk. However, they can also be among the metabolites of some ruminal or milk-contaminating microorganisms, [18,19]. In this case, the origin of indole and skatole (and related odors) can be both connected to cow’s metabolism or milk post-contamination. To sum up, several molecules have been identified as potential key-flavor compounds of grass-based milk and dairy products, but their real sensory impacts still need to be defined in detail.

Even though the knowledge on milk flavor is still in progress, the existence of aromatic differences between grass-based and non-grass-based milk is often evident. In general, milk from total mixed ratio-fed cows is described as malty, milky and sweet, whereas grass feeding is associated with animal, stable, green vegetable and medicinal aroma [20,21,22]. It is mostly believed that such differences are primarily caused by different concentrations of a common set of flavor compounds rather than by the occurrence of compounds uniquely associated with a particular feed [23]. In fact, aroma-active compounds exclusive of milk from specific feeding regimen seem to be rare: all molecules mentioned above as possible components of grass-based milk aroma are also found in the other types of milk, but at lower concentrations. An aspect that needs to be fully clarified is the “weight” of the different sources of aroma-active compounds: cows’ metabolism and direct-transfer from feed. If the former source is predominant, finding specific connections with the territory (intended as the type of plants eaten by cattle) becomes very difficult. It is the opposite in the case of higher weight of the direct transfer mechanism: in this perspective, another variable may come into play, that is the pulmonary route. Essentially, it is a possibility that the volatile compounds inhaled by animals from the surrounding environment are transferred to blood and then to milk. This aspect is increasingly attracting the interest of the scientific community and the first outcomes are very interesting [7,24]. If the impact of the pulmonary route will be ascertained as highly significant, a new horizon could open in the dairy sector, fortifying the importance of animal welfare and helping to safeguard dairy farming in marginal but pristine areas.

If a clear picture of the flavor of grass-based milk has not yet been defined, the knowledge on transformed products can only be worse. Of course, the most investigated transformed product is cheese. Under the economic point of view, connecting the flavor of a cheese to the territory in which it has been produced and, possibly to animals’ welfare, throughout the influence of cattle feeding is a powerful tool to stand out from the competition. As mentioned above, if we specifically refer to taste and aroma, much work is still needed to scientifically support such a claim. For sure, the most complicated is the manufacturing process of the cheese, the most difficult is to find connections with the flavor characteristics of the milk used. It is the case, for instance of pasta filata cheeses, which undergo a strong heating treatment during the stretching phase [20], and of Blue cheeses, whose manufacturing process involves the use of primary and secondary selected starters (the molds) which are responsible for the intense formation of highly impacting aroma compounds. In these cases, the link with the feeding regimen should be searched by looking at the peculiar composition of the flavor precursors, first of all triglycerides, whose level of unsaturation should have an influence of the qualitative–quantitative pattern of the volatile compounds formed during processing and ripening.

In conclusion, the current knowledge provides robust scientific evidence for the assertion that the flavor of grass-based dairy products is different from that of non-grass-based ones. However, much work is still needed to associate the differences to specific compounds and to the sources from which they originate. At this time, we absolutely share the opinion expressed in the above-mentioned work of Kilcawley et al. [5] that “… consumers appear less able to discriminate sensory differences in milk and cheese than trained assessors and that differences in visual (mainly colour) and textural attributes were easier to discern that flavour attributes. This likely suggests that sensory differences due to diet are often subtle”.
